# A recovered Moho model by integrated inversion of gravity and seismic depths in Iran

**DOI:** 10.1016/j.heliyon.2020.e03636

**Published:** 2020-03-24

**Authors:** Sahar Ebadi, Abdolreza Safari, Riccardo Barzaghi, Abbas Bahroudi

**Affiliations:** a School of Surveying and Geospatial Engineering, College of Engineering, University of Tehran, Tehran, Iran; b Department of Civil and Environmental Engineering, Politecnico di Milano, Milan, Italy; c School of Mining Engineering, College of Engineering, University of Tehran, Tehran, Iran

**Keywords:** Geophysics, Moho, Gravimetric inverse problem, Integrated inversion, Collocation, Seismic depths

## Abstract

This research aims to define the depth of Moho in Iran by collocation method using gravimetric data with seismic information. The definition of the Moho in the Iranian region is of considerable importance due to the geological complexity of the area also characterized by tectonic and orogenic events of particular uniqueness. We applied the collocation method to Moho recovery using the gravity data generated by GOCO03S model reduced by topography/bathymetry, sediment and consolidated crust effects from CRUST1.0. These data have been complemented with seismic Moho depth estimates. A compilation of 213-points seismic depth has been collected over Iran and used in the integrated gravimetric-seismic inversion. Among them, 140 seismic points have been selected completely random and included as data in the integrated collocation approach for Moho depth estimation. The 73 remaining seismic points have been used as checking points for validating the estimated Moho. In the first run, gravity data only have been considered to collocation Moho recovery. When comparing this gravimetric solution with the 73 seismic checking points, a standard deviation of 6.2 km was found. In case of considering the regional seismic depths into the collocation approach, the standard deviation of the residuals between our results and seismic checking Moho depths improved to 4.9 km. It must be stated that, even in the integrated inversion, a significant discrepancy between the seismic and the integrated gravimetric-seismic Moho is present in the South Caspian Basin. Low quality of CRUST1.0 could explain this inconsistency in this area.

## 1 Introduction

The Moho interface is defined as the transition layer separating the lowermost crust from the underlying mantle (Turcotte and Schubert, 1982). Moho mapping can be obtained via a variety of geophysical investigations like seismic refraction and reflection studies and gravity inversions (see, e.g. (Parker, 1973; Beloussov et al., 1980; Braile and Chiang, 1986; Lebedev et al., 2013)). Since the seismic Moho coverage is spatially limited, we can profitably use the result of gravimetric studies. Moreover, the advent of satellite dedicated gravity missions (Reigber et al., 1999; Tapley et al., 2004; Floberghagen et al., 2011), made possible to estimate the Moho depth at global scale. Eshagh et al. (2011) and Reguzzoni et al. (2013) proposed methods that optimally combine seismic and gravimetric data for Moho estimate.

The estimation of the Moho discontinuity in Iran is one of the most critical issues for the geoscience community. The convergence of the Arabia-Eurasia Plate during the Mesozoic and Cenozoic period led to the complex features in this region (Berberian and King, 1981; Berberian et al., 1982; Mouthereau et al., 2012). Due to this continental collision, the Iranian plateau is characterized by some active and young tectonic structures including the collision zones in Zagros, Alborz and Kopeh-Dagh and the subduction zones in the Makran and South Caspian Basin. Extensive investigations into the crustal thickness of the Iranian plateau have been carried out based on some geophysical surveys (Mangino and Priestley, 1998; Paul et al., 2006; Taghizadeh-Farahmand et al., 2010, 2015; Radjaee et al., 2010, Shad Manaman et al., 2011; Tatar and Nasrabadi, 2013; Motaghi et al., 2015; Abdollahi et al., 2018). Also, Eshagh et al. (2017) and Ebadi et al. (2019) estimated the Moho depths in the Iranian region inverting gravity data.

In this paper, we applied the collocation inversion method for a two-layer model devised by Barzaghi and Biagi (2014) to Moho estimate over Iran. They implemented an updated version of the collocation method (Krarup, 1969; Moritz, 1990; Barzaghi et al., 1992, 2015), which allows the integration of the gravity and seismic derived depths. This approach is based on the propagation of the covariance structure the Moho depth to the covariance of the gravity data. According to this method, gravity observations and the seismic Moho depth information were combined, and the integrated gravimetric-seismic Moho estimate was obtained in the Iran study area. The gravity has been generated from GOCO03S gravitational model (Mayer-Gürr et al., 2012) corrected for the SRTM30_PLUS topographic/bathymetric data (Becker et al., 2009) and the sediment and the crystalline data of CRUST1.0 (Laske et al., 2013). The seismic Moho depths used in the integrated inversion have been collected from the available literature.

The paper is conceived as follows: In Section 2, the collocation method is revised and the basic formulas applied in the computations are derived. Section 3 is dedicated to the application of the collocation inversion method to the Iran case study while in Section 4, comments and conclusions are reported.

## 2 The theoretical background of the collocation solution

In the following, the collocation procedure is presented as a regularization technique to solve the gravity inversion problem for Moho recovery (see, e.g. (Backus and Gilbert, 1967; Jackson, 1979)). We adopted this method in a two layers model with known density contrast. The collocation method allows considering the seismic Moho depths as a priori information to constrain the Moho solution. The basis of this method is the propagation of the covariance structure of the Moho depth to the covariance of the measured gravity in the adopted simple two-layer model (Barzaghi et al., 1992, 2015; Barzaghi and Biagi, 2014).

In planar approximation, the basic formula that gives the linearized relationship between gravity and Moho depth is (Barzaghi and Biagi, 2014):

(2-1) $$\Delta g\left(x,y,0\right)=G\iint_{R^{2}}dxdy\Delta \rho \frac{\varepsilon T_{0}}{{\left[T_{0}^{2}+d_{xy}^{2}\right]}^{3/2}}$$

In this equation, Δg is the Bouguer gravity anomaly minus its mean, *G* stands for the Newton's gravitational constant, $T_{0}$ is the mean Moho depth, ε is the anomalous depth respect to $T_{0}$, Δρ is the mean constant density contrast between the two layers and $d_{xy}=\sqrt{x^{2}+y^{2}}$.

In order to get an integrated estimate of Moho depth, the gravity data and seismic Moho depths can be used together. The observed gravity values and the Moho depths, containing the respective noise components, can be written as:

(2-2) $$\Delta {\text{g}}_{OBS}=\Delta \text{g}+{\text{n}}_{g}$$

(2-3) $${\text{ε}}_{OBS}=\text{ε}+{\text{n}}_{\varepsilon }$$

All of the collocation formulas are then derived under the following hypotheses (Barzaghi and Biagi, 2014):

1) ε is a weak stationary stochastic process, ergodic in the mean and in the covariance
2) The noises in gravity and depth, $n_{g}$ and $n_{\varepsilon }$ are spatially uncorrelated zero mean signals
3) The cross-correlations between signals and noises are zero

Based on the given assumptions, the auto and cross-covariances between gravity and depth are:

(2-4) $$C\left(\Delta g_{i},\Delta g_{j}\right)=C_{\Delta g\Delta g}\left(\left|P_{i}-P_{j}\right|\right)=C\left(\Delta g_{j},\Delta g_{i}\right)$$

(2-5) $$C\left(\varepsilon_{i},\varepsilon_{j}\right)=C_{\varepsilon \varepsilon }\left(\left|P_{i}-P_{j}\right|\right)=C\left(\varepsilon_{j},\varepsilon_{i}\right)$$

(2-6) $$C\left(\varepsilon_{i},\Delta g_{j}\right)=C_{\varepsilon \Delta g}\left(\left|P_{i}-P_{j}\right|\right)=C\left(\Delta g_{j},\varepsilon_{i}\right)$$

(2-7) $$C\left(n_{i},n_{j}\right)=\delta_{ij}\sigma_{i}^{2};\;C\left(n,\Delta g\right)=C\left(n,\varepsilon \right)=0$$

The collocation estimate of ε can be obtained according to two schemes; inverting only the gravity data and applying integrated inversion of the observed gravity values and seismic derived depths. When using the gravity data only, the estimator of collocation can be written as (Moritz, 1980; Barzaghi et al., 1992; Barzaghi and Biagi, 2014):

(2-8) $$\hat{\varepsilon }=\left[{\text{c}}_{\varepsilon \Delta g}^{T}\right]{\text{C}}_{\mathrm{ll}}^{-1}\text{l}$$

With ${\text{c}}_{\varepsilon \Delta g}=C\left(\varepsilon_{k},\Delta {\text{g}}_{OBSi}\right),\;1=\left[\Delta {\text{g}}_{OBS}\right]\;\text{and}\;C_{ll}=\left[C_{\Delta g_{OBS}\Delta g_{OBS}}\right]$.

When the observed seismic depths are included as input data as well as the gravity values, the ε is given by (Moritz, 1980; Barzaghi et al., 1992; Barzaghi and Biagi, 2014):

(2-9) $$\hat{\varepsilon }=\left[{\text{c}}_{\varepsilon \Delta g}^{T}{\text{c}}_{\varepsilon \varepsilon }^{T}\right]{\text{C}}_{ll}^{-1}\text{l}$$

With ${\text{c}}_{\varepsilon \Delta g_{i}}=C\left(\varepsilon_{k},\Delta {\text{g}}_{OBSi}\right),\;{\text{c}}_{\varepsilon \varepsilon_{i}}=C\left(\varepsilon_{k},\varepsilon_{OBSi}\right),\;\text{l}=\left[\begin{array}{c} \Delta {\text{g}}_{OBS}\\ \varepsilon_{OBS} \end{array}\right]\;\text{and}\;{\text{C}}_{ll}=\left[\begin{array}{cc} {\text{C}}_{\Delta g_{OBS}\Delta g_{OBS}} & {\text{C}}_{\Delta g_{OBS}\varepsilon_{OBS}}\\ {\text{C}}_{\varepsilon_{OBS}\Delta g_{OBS}} & {\text{C}}_{\varepsilon_{OBS}\varepsilon_{OBS}} \end{array}\right]=\left[\begin{array}{cc} {\text{C}}_{\Delta g\Delta g} & {\text{C}}_{\Delta g\varepsilon }\\ {\text{C}}_{\varepsilon \Delta g} & {\text{C}}_{\varepsilon \varepsilon } \end{array}\right]+\left[\begin{array}{cc} {\text{C}}_{nn_{\Delta \text{g}}} & \text{0}\\ \text{0} & {\text{C}}_{nn_{\varepsilon }} \end{array}\right]$.

In order to compute this estimate, we first have to define the empirical covariance function of $\Delta g_{OBS}$ and fit it with appropriate positive definite model functions (Moritz, 1980). The empirical covariance of the gravity data can be estimated as (Barzaghi et al., 1992):

(2-10) $$\begin{array}{l} {\hat{C}}_{\Delta g\Delta g}\left(\Delta P_{k}\right)=\frac{1}{N}\sum_{i=1}^{N}\frac{1}{N_{i}}\sum_{j=1}^{N_{j}}\Delta g_{OBS}\left(Q_{i}\right)\Delta g_{OBS}\left(Q_{j}\right)\\ P_{k-1}<\left|Q_{i}-Q_{j}\right|<P_{k},\;\;\Delta P_{k}=P_{k}-P_{k-1} \end{array}$$

The auto and cross-covariance models are then needed to get the ε for Moho interface estimate. A suitable covariance model for the auto-covariance of Δg (see Barzaghi and Biagi (2014)) can be:

(2-11) $$C_{\Delta g\Delta g}\left(r\right)=\frac{AJ_{1}\left(\alpha x\right)}{\alpha x}$$

where $J_{1}\left(\cdot \right)$is the first order Bessel function.

Considering this auto-covariance, one can prove that (Barzaghi et al., 1992):

(2-12) $$C_{\varepsilon \Delta g}\left(P,Q\right)=\frac{A}{2\pi G\Delta \rho \alpha }\int_{0}^{\alpha }dkke^{kT_{0}}J_{0}\left(k\left|P-Q\right|\right)$$

(2-13) $$C_{\varepsilon \varepsilon }\left(P,Q\right)=\frac{A}{{\left(2\pi G\Delta \rho \alpha \right)}^{2}}\int_{0}^{\alpha }dkke^{kT_{0}}J_{0}\left(k\left|P-Q\right|\right)$$

where $J_{0}\left(\cdot \right)$ is zero order Bessel function.

The values of the two functions in (2-12) and (2-13) can be computed by using numerical integration methods while the A and α values are estimated by fitting the model (2-11) into the empirical estimated covariance values of Δg (Barzaghi and Sansò, 1983).

By deriving $C_{\varepsilon \Delta g}\left(P,Q\right)$ and $C_{\varepsilon \varepsilon }\left(P,Q\right)$ from the expression above and inserting them in (2-8) and (2-9), theε value can be obtained. The final Moho depth is given respect to the mean depth as $T=T_{0}+\varepsilon$. A refined estimate can be determined by iteration so that the final solution is obtained as $T=T_{0}+\varepsilon_{1}+...+\varepsilon_{n}$.

## 3 The Iran case study

The methodology reviewed in Section 2 was applied in this section to Moho determination in the study area of Iran. This section is divided into four main parts. The geological classification of Iran block are characterized in section 3.1 and the used gravity data are described in section 3.2. We then present the Moho estimate over the study area by collocation approach in section 3.3. Finally, in section 3.4, we compared the Moho solutions from collocation method with some seismic derived Moho values of the area.

### 3.1 The study area

Due to the convergence of the Arabian-Eurasian plate during the geological times, Iranian crust and lithospheric mantle are characterized by the complex structures. The occurrence of the closure of Tethys Ocean and collision of Arabian-Eurasian plates led to the formation of Iranian plateau during the Mesozoic and Cenozoic period (Berberian and King, 1981; Berberian et al., 1982). There are active and young tectonic structures within Iranian plateau including the collision zones in Zagros, Alborz, Kopeh-Dagh and subduction zones in the Makran and South Caspian Basin (Shad Manaman et al., 2011). Moreover, two tectonometamorphic and magmatic belts of Sanandaj-Sirjan zone and the Urumieh-Dokhtar magmatic assemblage are the results of the collision of Arabian and Eurasia plates. Central Iran is known as a triangular area in the middle and limited to the Alborz Mountains in the North, Lut Block in the East and Urumieh-Dokhtar in the South. This region consists of different rocks from all ages, from Precambrian to Quaternary, and several episodes of orogeny, metamorphism and magmatism. Sanandaj-Sirjan is situated in the South-West of Central Iran and the North-East of Zagros Mountains. The presence of immense volumes of magmatic and metamorphic rocks of Paleozoic and Mesozoic eras is the main feature of this zone. The Arabian Block is separated from the rest of the Eurasian tectonic plate by Zagros ranges, which is without magmatic and metamorphic events. Alborz Mountain is located in North of Iran, parallel with the Southern border of Caspian Sea. This range is composed of different sedimentary rocks. The Kopeh-Dagh Mountains and basin includes mainly extrusive igneous rocks belong to Paleogene volcanic areas. A long-range of ophiolites extending from West to East led to separate Makran from Jazmourian depression (cf. Ghorbani (2013)).

**Figure 1:**
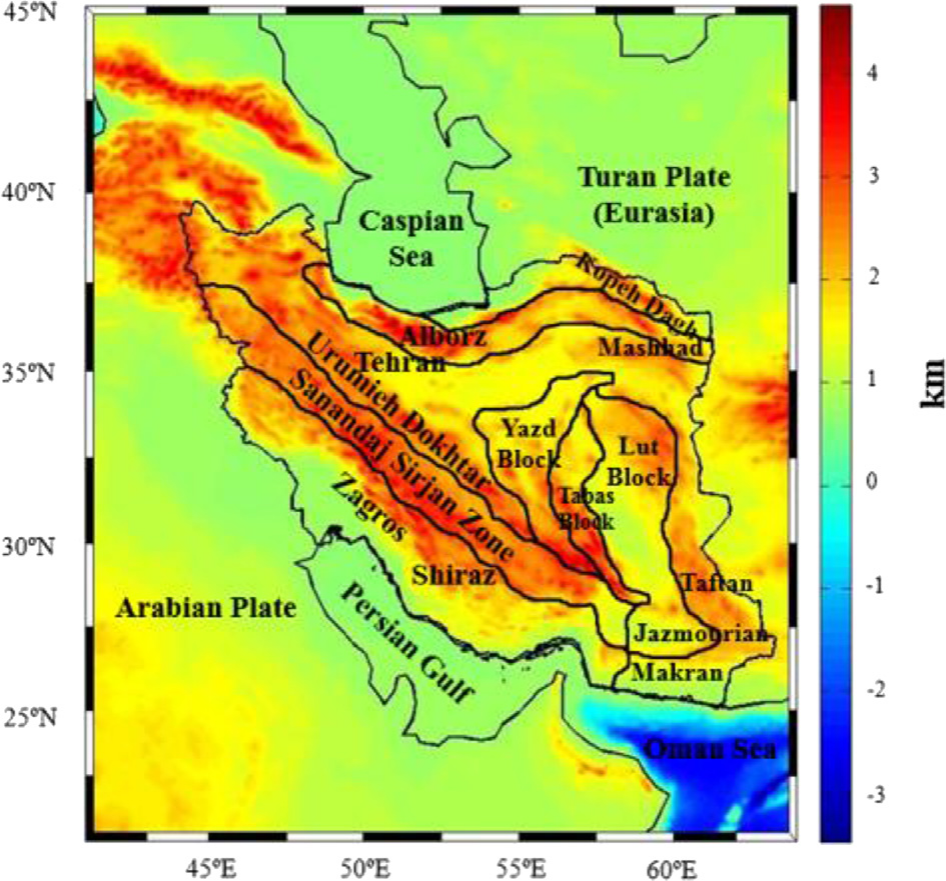
Topography heights and geological setting of the study area of Iran [km].

The regional study area of Iran is limited by the parallels 20° and 45° North and the meridians 40° and 65° East. As already pointed out, this area is characterized by complicated structural units because of several unique events like tectonics and orogenic activities. Some models and interpretations have been suggested for the geological structure of the Iran block (Nabavi, 1976; Alavi-Naini, 1993; Aghanabati, 2004; Ghorbani, 2013). According to these investigations, the study area of Iran has been geologically classified into various structural zones. This geological classification is superimposed on the regional topography and shown in Figure 1. We applied SRTM30_PLUS model to generate the topographic/bathymetric heights to degree and order 2160 with a resolution of 5' × 5′ over the study area (Becker et al., 2009). This regional map shows a significant topography over the Alborz and Zagros mountains with height values ranging from -3182 to 4142 m. This clearly shows the roughness of the Iranian topography, which has smooth feature only in the southern border of Caspian Sea, Central Iran, Lut Block, Jazmourian and Makran basins.

### 3.2 The gravity data set

**Figure 2:**
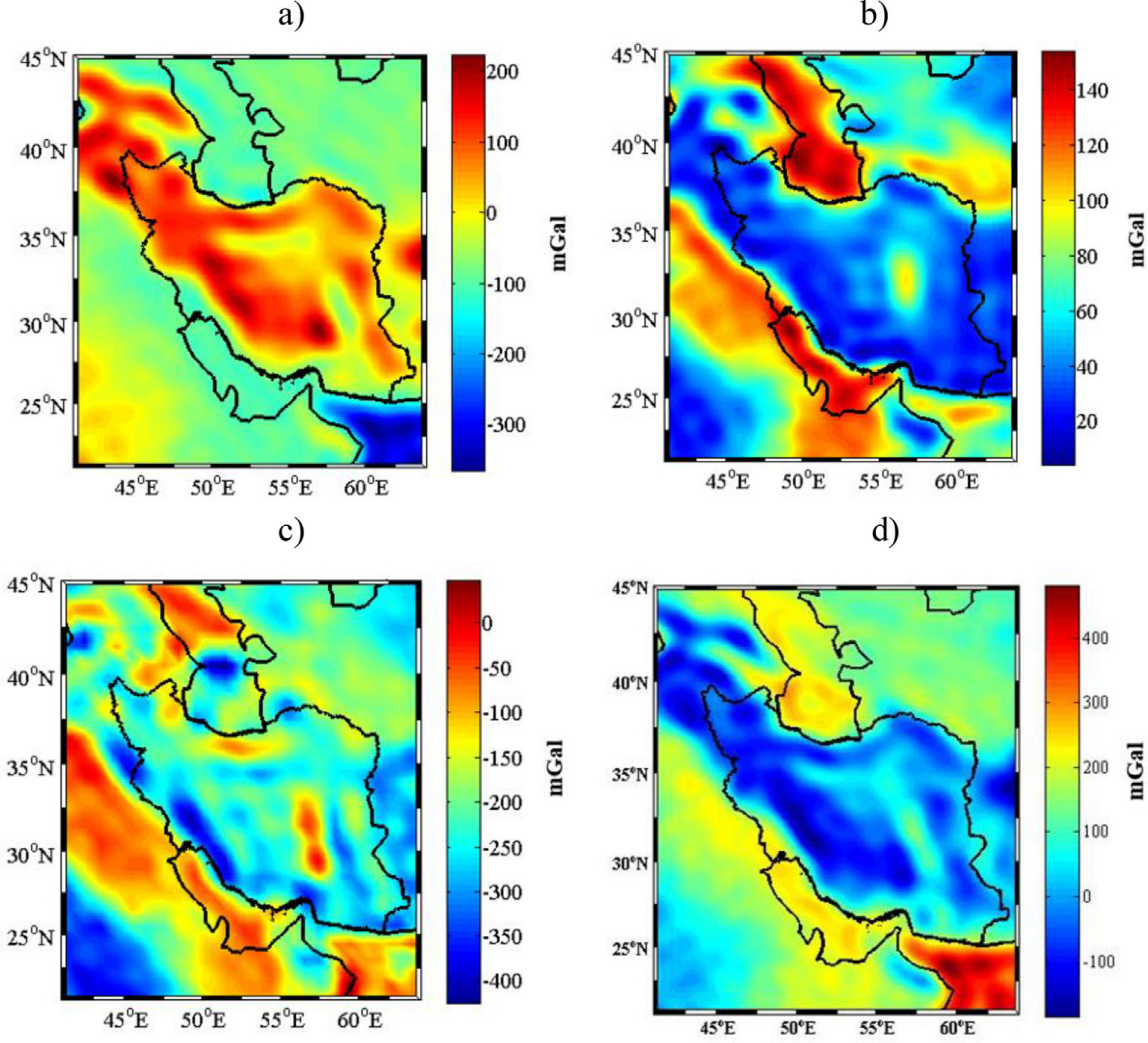
a) the TB gravitational effect; b) the gravitational effect of sediments; c) the gravitational effect of consolidated crust; d) the corrected Bouguer gravity anomalies [mGal].

The gravity data of the study area were retrieved from GOCO03S global gravitational model up to degree and order 180 and were computed on a 0.5×0.5 arc-deg surface grid. We used the coefficients of the digital elevation model from SRTM30_PLUS to degree and order 180 to generate the Topography/Bathymetry (TB) corrections. In order to obtain the gravity corrections due to sediments and consolidated crust, we applied the Earth's crustal model CRUST1.0 (see Figures 2b and 2c), with a spectral resolution up to degree 180. The refined gravity data that were used in collocation procedure were obtained from applying all of these corrections. The map of the refined gravity data has been represented in Figure 2d, in a unit of mGal. The largest and positive contribution of these effects on gravity data can be seen at Oman Sea, and the negative one is over the Zagros and Sanandaj-Sirjan belts and in the North-East part of Iran around its border to Azerbaijan and Turkey and in the south part in Bam. All the mentioned corrections and the resulting refined gravity data are shown in Figure 2, while the related statistics are given in Table 1.

**Table 1:** Statistics of the TB, sediment and consolidated crust corrections to gravity anomalies [mGal].

	Max	Mean	Min	STD
TB	227.3	-35.0	-371.1	96.7
sediments	183.5	66.0	3.9	34.5
crust	46.5	-199.5	-424.7	84.2
total	486.8	230.6	-195.1	123.2

### 3.3 The collocation Moho estimate

The formulas described in section 2 were applied to the determination of Moho depth in the study area by using an in-house developed software.

In performing the inversion, the constant value of Δρ = 600 kg m^−3^ for the density contrast between crust and mantle has been assumed. Furthermore, the mean Moho depth has been fixed to $T_{0}=42$ km, i.e. the mean depth of the seismic Moho values over Iran (Mangino and Priestley, 1998; Paul et al., 2006; Taghizadeh-Farahmand et al., 2010, 2015; Radjaee et al., 2010; Tatar and Nasrabadi, 2013; Motaghi et al., 2015; Abdollahi et al., 2018).

**Figure 3:**
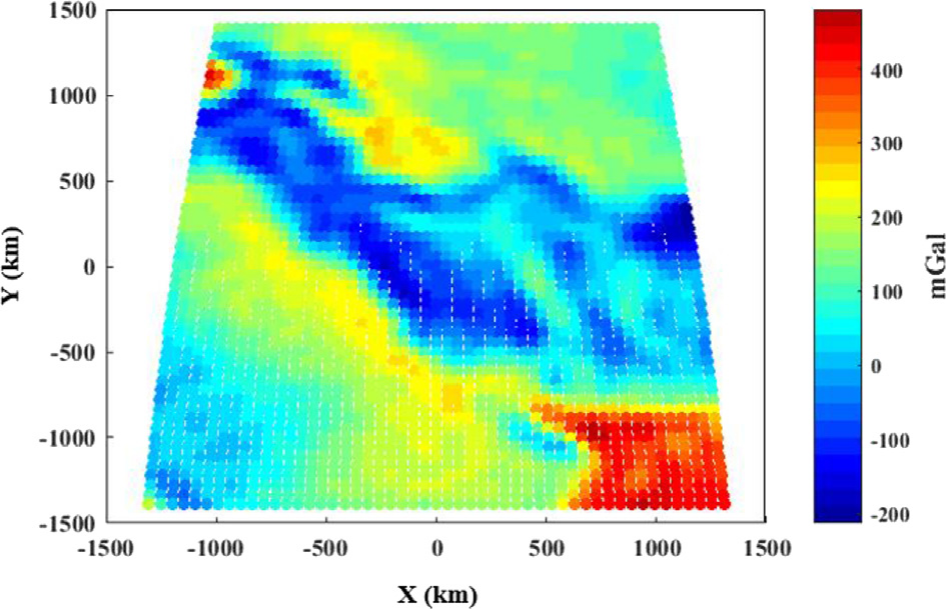
Bouguer gravity anomaly corrected by topography/bathymetry, sediment and consolidated crust corrections on a (x,y) grid [mGal].

In order to use the collocation approach in Moho recovery, the corrected gravity data should be re-gridded on a regular (x,y) grid in the investigation area, as shown in Figure 3:

The computations were performed according to two schemes; gravity data only and integrated inversion of gravity and seismic derived Moho depths. In order to estimate the integrated gravimetric-seismic Moho, we have selected 140 seismic points that were used in the collocation procedure together with the gravity data.

**Figure 4:**
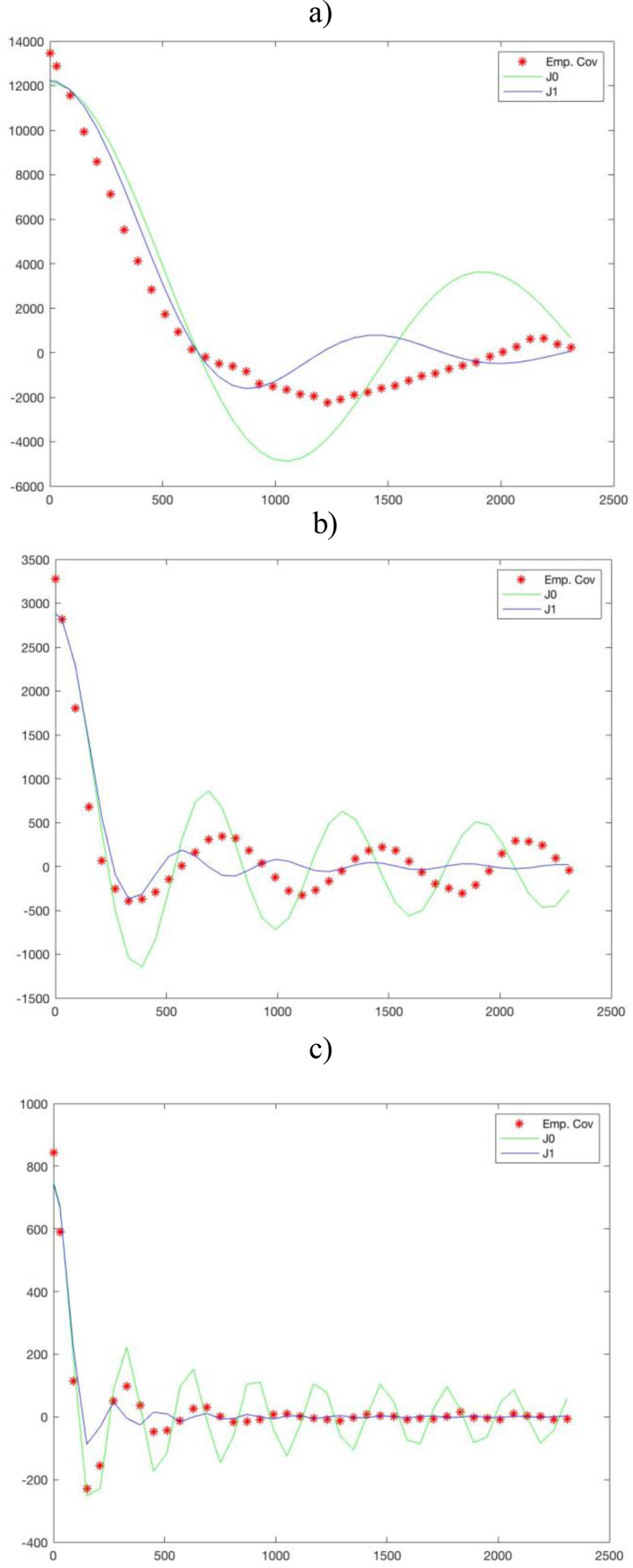
The empirical covariance function of the gravity data and the best-fit model a) First iteration step: ${\hat{A}}_{\delta g}=$12232 mGal^2^, $\hat{\alpha }=$0.0058 km^−1^, ${\hat{\sigma }}_{n}^{2}=$1227.7 mGal^2^ b) Second iteration step: ${\hat{A}}_{\delta g}=$2886 mGal^2^, $\hat{\alpha }=$0.015 km^−1^, ${\hat{\sigma }}_{n}^{2}=$387.9 mGal^2^ c) Third iteration step: ${\hat{A}}_{\delta g}=$745 mGal^2^, $\hat{\alpha }=$0.031 km^−1^, ${\hat{\sigma }}_{n}^{2}=$98.8 mGal^2^.

As already mentioned, we applied the collocation approach as an iterative process. Figure 4 illustrates the behavior of the empirical covariance function of the original gravity data and the best-fit model with the relevant parameters when inversion is performed using gravity data only. It has to be mentioned that the collocation solution depends to the second order on the covariance (Sansó et al., 2000). Thus, the fit between the empirical values and the model is not so critical, particularly if this refers to the empirical values at large steps, where the empirical estimates are less reliable (Sansò, 1985).

In Figure 4, the *A* and αparameters represent the covariance value in origin and the scaling factor for the argument of model covariance in (2-11), respectively. The ${\hat{\sigma }}_{n}^{2}$ is the noise variance, which stands for the difference between the empirical value and the model function at the origin. To find the *A* and α values, the first empirical zero is set to coincide with the zero of the model function, further assuming that the model function coincides with the empirical one at the second point (Barzaghi et al., 1992). In the first step, the *J*_*1*_ Bessel function with arguments of *A* = 12232 mGal^2^ and $\hat{\alpha }$ = 0.0058 km^−1^ is the model function that better describes the empirical values. The covariance value in origin from the model function is sufficiently close to the initial one of empirical function, which is 13460 mGal^2^. The variance noise defined by the difference between the two values in origin has been evaluated in 1227.7 mGal^2^. The residuals of gravity from the first step have been considered as input data in the second step. As seen in Figure 4, the value in origin of the empirical function reduced drastically to 3273 mGal^2^ and the signal variance, i.e., the *A* value, has been fixed at 2886 mGal^2^. The $\hat{\alpha }$ and ${\hat{\sigma }}_{n}^{2}$ parameters have been set to 0.015 km^−1^ and 387.9 mGal^2^, respectively. In the third step, model covariance parameters were fixed at ${\hat{A}}_{\delta g}=$745 mGal^2^, $\hat{\alpha }=$0.031 km^−1^, ${\hat{\sigma }}_{n}^{2}=$98.8 mGal^2^ according to the residual gravity from the second iteration.

Similar plots and considerations hold when using gravity and seismic depths.

Both for the pure gravimetric and the integrated inversion, the iterative process has been stopped after the third step because the covariance function of the third iteration step residuals has a correlation length (the distance at which the covariance function is half of its value in origin) that is comparable with the grid step.

**Figure 5:**
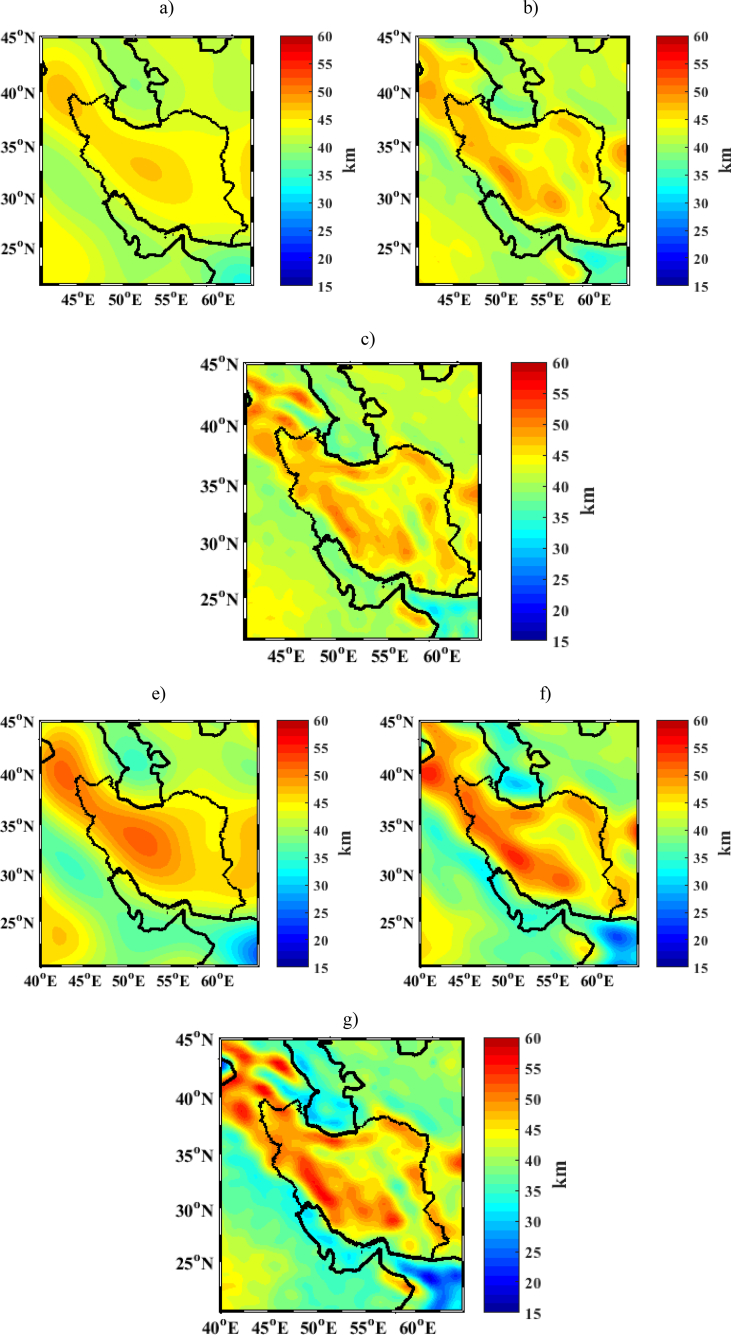
Map of Moho model derived from collocation method in Iran a) First step using gravity data only b) Second step using gravity data only c) Third step using gravity data only e) First step by integrated inversion of gravity and seismic depths f) Second step by integrated inversion of gravity and seismic depths g) Third step by integrated inversion of gravity and seismic depths [km].

The final gravimetric solutions are shown in Figures 5a, 5b, and 5c (first, second, and third iteration respectively) while the integrated gravimetric-seismic inversion is illustrated in Figures 5e, 5f, and 5g (first, second, and third iteration respectively). The statistics of these solutions are given in Table 2.

**Table 2:** Statistics of Moho depth computed according to collocation method using gravity data only and integrated inversion of gravity and seismic depths [km].

	Max	Mean	Min	STD
using gravity data only - first iteration step	47.5	42.6	34.1	2.4
using gravity data only - second iteration step	50.4	42.6	31.4	3.0
using gravity data only - third iteration step	53.1	42.6	30.2	3.4
integrated inversion of gravity and seismic depths first iteration step	52.0	42.6	24.3	4.6
integrated inversion of gravity and seismic depths second iteration step	55.7	41.6	23.5	5.5
integrated inversion of gravity and seismic depths third iteration step	58.0	40.7	18.8	6.2

The collocation estimate using gravity data only varies between 30.2 km and 53.1 km after three iteration steps while in the integrated inversion of gravity and seismic depths, the Moho depth ranges between 18.8 km and 58.0 km. As expected, in both cases, the iterative process led to a Moho estimate containing higher frequency patterns, which reflects the increase in the standard deviation of the estimated values. Furthermore, as can be seen in Figure 5, the pure gravimetric Moho is smoother than the estimate obtained by use gravity and seismic depths. The maximum Moho depth is under the Zagros Mountain, the Sanandaj-Sirjan and the Urumieh-Dokhtar belts, the Alborz Mountains and Kopeh-Dagh, whilst very smooth Moho is seen under the Oman Sea and the border of Caspian.

### 3.4 Evaluation of the Moho estimates

**Figure 6:**
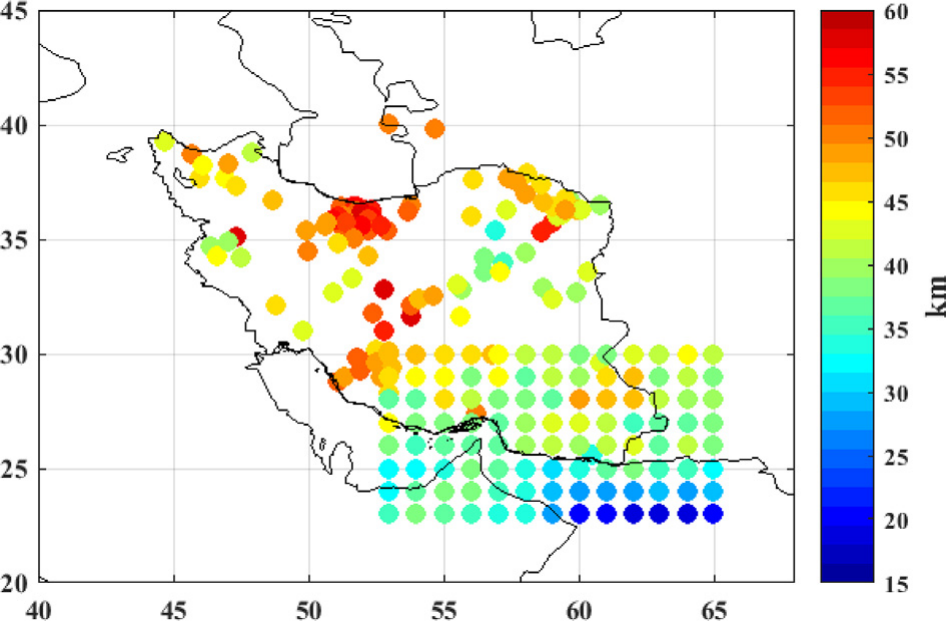
Moho derived from seismic results [km].

The Moho solution from collocation method is validated using existing regional seismic studies for Iran (Mangino and Priestley, 1998; Paul et al., 2006; Taghizadeh-Farahmand et al., 2010, 2015; Radjaee et al., 2010; Tatar and Nasrabadi, 2013; Motaghi et al., 2015; Abdollahi et al., 2018). We have compiled a 213-points collection of the seismic Moho datasets in the Iran area, and we have checked their consistency. These data are shown in Figure 6 and their statistics are summarized in Table 3. The Moho deepening derived from seismic studies ranges between 18.5 km and 58 km. As seen in Figure 6, the maximum Moho depth is located under the Zagros Mountains, and the Sanandaj-Sirjan belts and the minimum depth of Moho is detected under the Oman Sea and Makran subduction zone.

**Table 3:** Statistics of Moho depth from Seismic estimates [km].

	Max	Mean	Min	STD
Seismic depths	58.0	42.5	18.5	8.2

**Figure 7:**
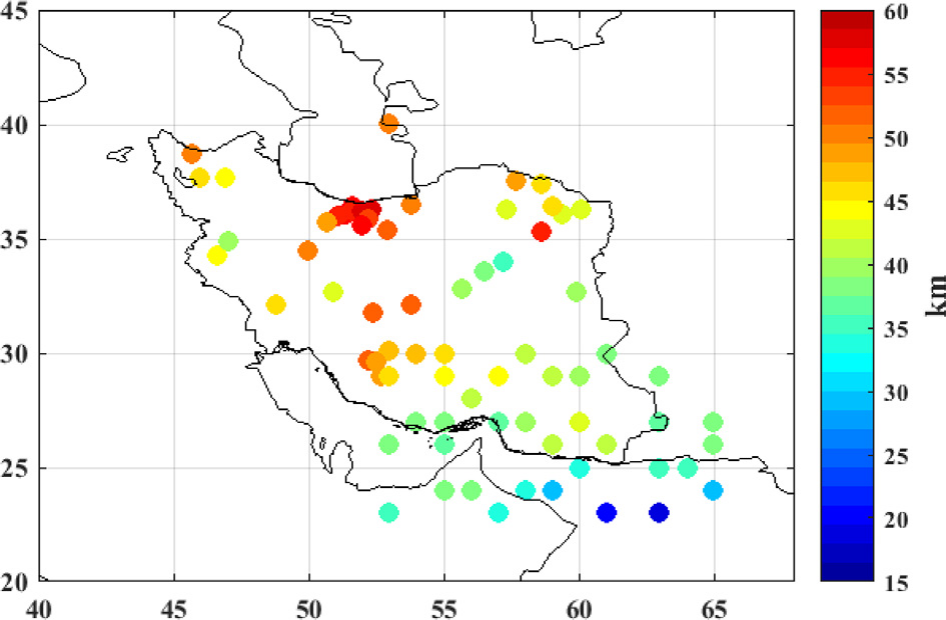
Moho checking points from seismic results [km].

As stated before, the collocation method has been applied according to the two schemes; using gravity data only and the integrated inversion of gravity and seismic depths. As explained in the previous section, in order to estimate the local Moho depth in the integrated inversion of gravity and seismic depths, we have selected 140 seismic depths completely random and used them in the collocation procedure. The evaluation of the results both using gravity only and the integrated inversion of gravity and seismic depths has been performed by comparison with the 73 remaining seismic points. The distribution of the selected seismic checking points is shown in Figure 7.

**Figure 8:**
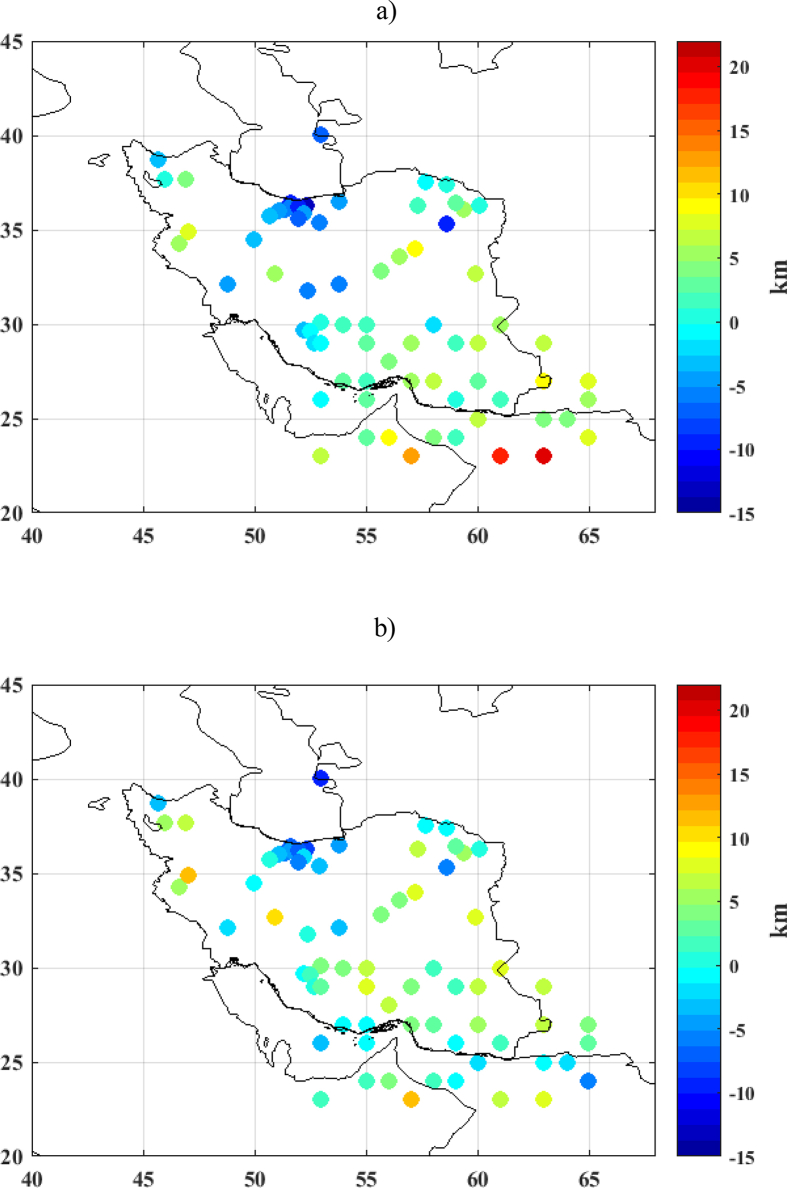
Differences between seismic depths and Moho derived from Collocation method a) by using gravity data only b) by integrated inversion of the gravity and seismic depths [km].

The differences between collocation solutions and 73 seismic checking points are shown in Figure 8 and the related statistics are summarized in Table 4. As can be seen in Figure 8a, the differences between the collocation solution using gravity data only and seismic depths ranges from -12.7 km beneath Alborz Mountains to 20.5 km under Oman sea bottom. When comparing the Moho estimate based on the integrated inversion of seismic depths and gravity data and the 73 seismic checking data points, the differences vary from -10.0 km under the northern part of Alborz Mountains to 11.8 km beneath the Sanandaj-Sirjan zone.

**Table 4:** Statistics of differences between Moho estimates from collocation method and seismic results [km].

	Max	Mean	Min	STD	RMS
using gravity data only	20.5	1.4	-12.7	6.2	6.3
integrated inversion of gravity and seismic depths	11.8	1.3	-10.0	4.9	5.1

As seen in Table 4, the comparison between collocation solutions and seismic Moho datasets indicates that the STD of differences decreases from 6.2 km to 4.9 km, when we applied integrated inversion of gravity and seismic depths rather than using gravity data only. Since the STD of seismic points is 8.2 km (see Table 3), it can be concluded that the collocation method can be reliably applied to Moho recovery when the integrated inversion of gravity and seismic depths is used. In this case, the solution has better statistics if compared to those of the pure gravimetric solution.

In order to have a deeper insight into the computed collocation solutions, these overall statistics can be further detailed for different sub-areas in the Iran region where existing regional seismic solutions are available.

In the area of the Iranian plateau, according to the investigations of Dehghani and Makris (1984), the crustal thickness ranges from 35 km beneath the Alborz Mountains to 54 km in central Alborz. In the same area, Radjaee et al. (2010) found a variable crustal at a depth between 55 and 58 km and a Moho thickness between 55 and 60 km has been proposed by Shad Manaman et al. (2011) beneath the central Alborz. According to the seismic checking points, the Moho depths ranges from 50 to 58 km under the Alborz Mountains. The integrated gravity and seismic collocation estimate is in good agreement with these estimates since it gives a crustal thickness around 54 km. Conversely, the collocation estimate based on gravity data only leads to a smaller thickness, around 48 km.

In another region, beneath the Kopeh-Dagh Mountains, Jiménez-Munt et al. (2012) reported a crustal thickness of 50 km. According to the map of seismic checking points in Figure 6, the Moho depth in this region ranges from 42 to 49 km. Again, the integrated gravity-seismic solution that we computed in this area is in good agreement with this estimate being around 50 km while the pure gravimetric estimate gives an underestimated value, around 47 km.

In the area of maximum Moho depth in Iran, i.e., the area under the Zagros Mountains (specifically Sanandaj-Sirjan zone), our computations using gravity data only indicates a 51 km Moho depth while applying the integrated gravity-seismic inversion depths up to 55 km are reached. This second estimate is in agreement either with the depths indicated by the seismic checking points that give Moho values ranging between 42 and 55 km in this region. Also, Dehghani and Makris (1984) estimated the Moho depth of 55 km by using integrated gravity and seismic depths beneath the central Zagros, which fully agrees with our integrated inversion results.

Thickness under Urumieh-Dokhtar magmatic area is estimated around 42 km according to Paul et al. (2006) while Taghizadeh-Farahmand et al. (2010) give the estimate of the crustal thickness around 48 km for this zone. In this area, the seismic checking points map shows values around 50 km beneath while the collocation solutions that we computed using gravity data only and integrated inversion of the gravity and seismic depths are between 44-47 km and 45–50 km, respectively. Thus, also, in this case, the integrated collocation estimates are in good agreement with the seismic values.

The variation of the crustal thickness has been proposed between 45 and 48 km in eastern Iran by Dehghani and Makris (1984). Shad Manaman et al. (2011) reported a Moho ranging between 35 and 40 km in central Iran and Lut block. According to Sadidkhouy et al. (2012), the Moho variations in Isfahan area are from 38.5 to 43 km. The seismic checking points map, shows Moho depths around 40 and 44 km in Lut block and central Iran, respectively. Our findings by using gravity data only and gravimetric-seismic Moho estimates both show the Moho depth in the range of 43 km in central Iran and 48 km in Lut block.

Mangino and Priestley (1998) estimated the Moho deepening of 30–33 km beneath the South Caspian Basin. Similar values have been reported for Moho depth in this area by Shad Manaman et al. (2011). Our results, both using gravity only and gravity-seismic depths, indicate that the average of Moho depth there is around 45 km. So, contrary to other areas, our findings are not in agreement with the seismic values, giving deeper Moho depths. This uneven result for this area could be related to the sediment and crystalline corrections computed using the CRUST1.0, as it is known from previous investigations this density model has low quality in marine areas (see Eshagh et al. (2017)).

In the coast of the Persian Gulf, a Moho depth of about 25 km has been suggested by Paul et al. (2006). Both of the collocation solutions and seismic checking points show the values around 35 km in this area. Shad Manaman et al. (2011) presented an increasing range of Moho from 25-30 km across the Oman seafloor and Makran subduction zone to 45–50 km in Taftan volcano. Taghizadeh-Farahmand et al. (2015) estimated depth of 35 km over this area. Abdollahi et al. (2018) gave values around 18–28 km for the Moho depth in Oman Sea. Our findings using gravity data only reveal the local Moho deepening around 30–35 km and 40–48 km in the Oman Sea and Makran subduction zone, respectively. Thus the pure gravimetric solution seems to be in disagreement with these seismic solutions. On the contrary, there is a relatively good agreement between the seismic values and the gravimetric-seismic solution, which gives values of 18.5–35 km and 40–44 km for Moho depth in Oman seafloor and Makran subduction zone, respectively.

Overall, these comparisons indicate that our findings from the integrated collocation inversion of gravity and seismic depths are in most cases in good agreement with the findings of the seismic investigations. On the contrary, the depths of the purely gravimetric collocation Moho are generally underestimated/overestimated in comparison with those derived by seismic.

## 4 Summary and concluding remarks

In this study, the collocation method for estimating the Moho in Iran has been applied both using gravity data only and gravity and seismic depths in an integrated inversion. The gravity data generated by GOCO03s satellite-only global geopotential model have been reduced by topography/bathymetry, sediment and crystalline crust data effects by using the SRTM30_PLUS DTM and the CRUST1.0 model. Also, a compilation of seismic depths consisting of 213 points has been collected both to be used in our solution and to evaluate the final results. In order to perform the integrated inversion of seismic and gravity data, 140 points out of the 213 were selected completely random and used in the integrated inversion procedure. Results were then validated over the remaining 73 seismic depths points. When estimating the Moho depths using the gravity data only, a smoother solution is obtained if compared to the integrated gravimetric-seismic Moho estimate. This reflects in the statistics of the differences between collocation estimates and seismic Moho values over the 73 checking points. When considering the integrated inversion estimate, better statistics are obtained over these checking points. Particularly, the standard deviation of the residuals drops to 4.9 km when using the integrated inversion solution as compared to 6.2 km, which is the standard deviation obtained when considering the pure gravimetric solution. Thus, when applying the integrated inversion of gravity and seismic depths, some high-frequency features of the Moho depth are recovered while a smoother estimate is obtained when collocation is applied to gravity data only.

The best agreement between our integrated solution and the seismic depths is found in Northeast of Iran, Lut Block, Central Iran, and Coast of the Persian Gulf. Most of the larger discrepancies have been detected over the collision zones (Zagros, Alborz) and South Caspian Basin. In the South Caspian Basin, this can be a side effect of the quite poor quality of the CRUST1.0 data in this area. Since they were used to reduce the observed gravity values, it is possible these values are biased, thus amplifying the differences between our estimations and the seismic values in this particular region. However, all in all, it can be stated that the integrated gravimetric-seismic collocation solution gave stable and reliable results and that this method can be used for interpolating in a physically consistent way, through gravity, seismic line information on Moho depths.

## Declarations

### Author contribution statement

S. Ebadi: Conceived and designed the experiments; Performed the experiments; Analyzed and interpreted the data; Contributed reagents, materials, analysis tools or data; Wrote the paper.

A. Safari, A. Bahroudi: Analyzed and interpreted the data.

R. Barzaghi: Conceived and designed the experiments; Analyzed and interpreted the data.

### Funding statement

This research did not receive any specific grant from funding agencies in the public, commercial, or not-for-profit sectors.

### Competing interest statement

The authors declare no conflict of interest.

### Additional information

No additional information is available for this paper.
